# *Pseudomonas aeruginosa serA* Gene Is Required for Bacterial Translocation through Caco-2 Cell Monolayers

**DOI:** 10.1371/journal.pone.0169367

**Published:** 2017-01-03

**Authors:** Masashi Yasuda, Syouya Nagata, Satoshi Yamane, Chinami Kunikata, Yutaka Kida, Koichi Kuwano, Chigusa Suezawa, Jun Okuda

**Affiliations:** 1 Division of Microbiology, Department of Medical Technology, Kagawa Prefectural University of Health Sciences, Kagawa, Japan; 2 Division of Microbiology, Department of Infectious Medicine, Kurume University School of Medicine, Fukuoka, Japan; East Carolina University Brody School of Medicine, UNITED STATES

## Abstract

To specify critical factors responsible for *Pseudomonas aeruginosa* penetration through the Caco-2 cell epithelial barrier, we analyzed transposon insertion mutants that demonstrated a dramatic reduction in penetration activity relative to *P*. *aeruginosa* PAO1 strain. From these strains, mutations could be grouped into five classes, specifically flagellin-associated genes, pili-associated genes, heat-shock protein genes, genes related to the glycolytic pathway, and biosynthesis-related genes. Of these mutants, we here focused on the *serA* mutant, as the association between this gene and penetration activity is yet unknown. Inactivation of the *serA* gene caused significant repression of bacterial penetration through Caco-2 cell monolayers with decreased swimming and swarming motilities, bacterial adherence, and fly mortality rate, as well as repression of ExoS secretion; however, twitching motility was not affected. Furthermore, _L_-serine, which is known to inhibit the D-3-phosphoglycerate dehydrogenase activity of the SerA protein, caused significant reductions in penetration through Caco-2 cell monolayers, swarming and swimming motilities, bacterial adherence to Caco-2 cells, and virulence in flies in the wild-type *P*. *aeruginosa* PAO1 strain. Together, these results suggest that *serA* is associated with bacterial motility and adherence, which are mediated by flagella that play a key role in the penetration of *P*. *aeruginosa* through Caco-2 cell monolayers. Oral administration of _L_-serine to compromised hosts might have the potential to interfere with bacterial translocation and prevent septicemia caused by *P*. *aeruginosa* through inhibition of *serA* function.

## Introduction

*Pseudomonas aeruginosa* is an opportunistic pathogen and a cause of infection-related mortality among immunocompromised patients in hospitals. Without any obvious non-intestinal infection, gut-derived sepsis in immunocompromised hosts frequently arises from invasion by high-virulence *P*. *aeruginosa* strains, particularly in the intestinal tract [1–6]. In addition, gut-derived sepsis occurs when an intestinal pathogen expressing virulence determinants encounters an appropriate host under stress conditions; for example, the PA-I lectin/adhesion, a binding protein of *P*. *aeruginosa*, plays a key role in lethal gut-derived sepsis in mice after a 30% hepatectomy, as bacterial strains lacking functional PA-I lectin/adhesion have an attenuated ability to adhere to and decrease the transepithelial electrical resistance (TEER) of cultured intestinal epithelial cell (Caco-2 cell) monolayers, as previously described in detail [7].

Hirakata and colleagues previously established an animal model of endogenous *P*. *aeruginosa* septicemia in neutropenic mice [1,8–10]. This experimental model recapitulates important steps in the infection process including bacterial adherence to human intestinal epithelial cells, disruption of the intestinal epithelial barrier, penetration through this barrier, invasion into the bloodstream, production of virulence factors, and death [8]. Furthermore, the pathological features of bacteremia in this model were similar to those of septicemia in human patients and consequently closely mimics the pathophysiology of septicemia in humans [1]. However, the mechanism through which *P*. *aeruginosa* adheres to and penetrates intestinal epithelial cells was unclear.

The human adenocarcinoma cell line Caco-2, although isolated from an adult human colon, is thought to be highly analogous to enterocytes of the fetal colon. TEER in Caco-2 cell monolayers grown on filters varies, depending on growth and the presence of tight junctions [11]. As previously described in detail [12], Hirakata *et al*. established a model using human intestinal Caco-2 epithelial cell monolayers to evaluate the adherence and penetration of *P*. *aeruginosa* strains; clinical isolates of *P*. *aeruginosa* from blood adhered to and penetrated intestinal Caco-2 cell monolayers to a greater extent than those isolated from sputum, which was accompanied by a drastic decrease in TEER [12]. In addition, an avirulent exotoxin A-deficient mutant of *P*. *aeruginosa* PAO1 did not cause a similar decrease in resistance. However, the molecular mechanism of *P*. *aeruginosa* adherence to Caco-2 cells and disruption of the epithelial barrier is still unclear. We attempted to specify genes associated with the penetration activity of *P*. *aeruginosa* strain PAO1 by constructing and exploiting a chromosomal transposon insertion mutant library to comprehensively understand the mechanism through which *P*. *aeruginosa* adheres to Caco-2 epithelial cells and disrupts the epithelial barrier.

A comprehensive transposon mutant library for *Pseudomonas aeruginosa* has been developed for creating saturating libraries of sequence-defined transposon insertion mutants in which each strain is maintained, through the use of *Escherichia coli* SM10*pir*/pIT2 (IS*lacZ*/hah insertions) [13]. The strategy for generating mutant libraries has enabled the comprehensive specification of genes required for a wide range of biological processes such as a clinically relevant antibiotic resistance in *P*. *aeruginosa* [14]. *P*. *aeruginosa* utilizes numerous cell-associated and extracellular virulence factors including lipopolysaccharides, flagella, pili, exotoxin A, elastases, proteases, type III secretion effectors (ExoS, ExoT, ExoY, and ExoU), pyocyanin, catalase, alginate, and LasR/RhlR quorum-sensing molecules [15,16]. However, critical factors that play a key role in penetration through the Caco-2 epithelial barrier are still largely unknown. In this study, to specify critical factors that are responsible for *P*. *aeruginosa* penetration through the epithelium, we isolated transposon insertion mutants with a marked reduction in penetration activity. Of these mutants, we focused on the *serA* mutant, as the association between this gene and penetration activity is unknown. In *E*. *coli* and *Corynebacterium glutamicum*, D-3-phosphoglycerate dehydrogenase (PGDH) activity of the SerA protein catalyzes the first committed step in the phosphorylated pathway of _L_-serine biosynthesis, and _L_-serine inhibits PGDH activity in an allosteric, cooperative manner [17–19]. In this study, we investigated whether the *serA* gene could be a key factor that mediates *P*. *aeruginosa* penetration through Caco-2 cell monolayers. Furthermore, to evaluate if *serA* is associated with PAO1 virulence, we used the insect infection model of *Drosophila melanogaster*, which was previously validated to evaluate the virulence traits of *P*. *aeruginosa* [20,21]. Here, we suggest that _L_-serine administrated orally to compromised hosts has the potential to interfere with bacterial translocation and prevent septicemia caused by *P*. *aeruginosa*, through inhibition of *serA* function.

## Materials and Methods

### Bacterial strains

*P*. *aeruginosa* strain PAO1, used to construct the IS*lacZhah*-tc insertion library, is our laboratory stock strain provided by Dr. Koichi Kuwano (Kurume University) [22]. *P*. *aeruginosa* strain PAO1 was also used as a standard strain that penetrates epithelial cell monolayers [12]. *E*. *coli* strain SM10λ*pir* carrying the plasmid pIT2 was kindly provided by Dr. Colin Manoil (University of Washington) [14]. *E*. *coli* DH5α strain was used as a control strain that does not penetrate epithelial cell monolayers [12] and was purchased from TOYOBO, Japan.

### Transposon mutagenesis of PAO1

Transposon mutagenesis was performed as described previously [23] with a slight modification; a 0.5 mL aliquot of *E*. *coli* λ*pir* (pIT2) culture grown in LB broth supplemented with 100 μg/mL of ampicillin was mixed with a 0.5-mL aliquot of PAO1 culture. The mixture was filtered with Whatman Nuclepore track-etched membranes (GE Healthcare, UK) and washed with 10 mM MgSO_4_. The filter was then removed from the apparatus, transferred to an LB agar plate, incubated at 37°C for 24 h to allow conjugation and transposition to occur, and then transferred to a test tube containing 1 mL of LB broth. Cells were removed from the filter by vortexing. The cells were plated on LB agar containing 128 μg/mL of tetracycline to select the growth of *P*. *aeruginosa* cells carrying transposon insertions, and 16 μg/mL of chloramphenicol to counter-select for *E*. *coli*. Individual colonies appeared after 2 d of incubation at 37°C. Overnight cultures of 5952 individual colonies were preserved at −80°C.

### Mutant screening

To screen for mutants with markedly reduced ability to penetrate Caco-2 cell monolayers, relative to penetration by the wild-type strain at 6 h post-infection, we performed a penetration assay using Caco-2 cell monolayers at a multiplicity of infection (MOI) of 100, as previously described [24]. An overnight culture of individual transposon insertion mutants that was grown to visible turbidity was inoculated onto Caco-2 cell monolayers on Transwell^®^ plates (Corning, USA) prepared at a density of 6 × 10^6^ cells per mL, as previously described [25].

### Penetration assay

We performed a penetration assay using Caco-2 cell monolayers on Transwell^®^ (Corning) plates at a MOI. of 100 as described previously herein. _L_**-**serine (Nacalai Tesque, Japan) was added at the indicated concentration to both the apical and basolateral sides of the Transwell^®^ plates and pre-incubated at 37°C for 15 min before addition of the overnight culture. The assay was performed in triplicate, and the results are expressed as the mean ± SD. *P*. *aeruginosa* PAO1 and *E*. *coli* DH5α were used as positive and negative controls, respectively.

### Specification of transposon insertion site

A detailed schematic representation of arbitrary PCR methodology for specification of mutated gene in transposon insertion mutants is available online (https://pga.mgh.harvard.edu/Parabiosys/projects/host-pathogen_interactions/library_construction.php). As an example, we also show a schematic representation of *serA* gene specification in transposon insertion mutants (S1 Fig). Transposon insertion sites were specified using a two-round arbitrary PCR protocol [13,26] with a slight modification. The PCR primer sequences used for specification of the Tn5 insertion site are listed in Table 1, and a combination of forward and reverse primers used for arbitrary PCR are shown in Table 2. A touchdown PCR was carried out, as previously described [26]. In the first round of PCR, a primer (5-PIT2(10841) or Tn5-OE end(5929)-arb) specific to the transposon sequence was paired with a semi-degenerate primer with a defined tail (ARB1 or ARB1D). Total DNA from individual transposon insertion mutants was isolated from an overnight culture using the DNeasy Blood & Tissue Kit (QIAGEN, Germany). For the first round of arbitrary PCR, PCR mix containing 1× GoTaq buffer (Promega, USA), 2.5 μM dNTPs, 1.25 units of GoTaq polymerase, 200 nM of each primer, and 2 ng/μL of total DNA as template was used. Thermal cycling parameters were 95°C for 5 min, followed by five cycles of 95°C for 45 s, 30°C for 45 s, and 72°C for 1 min, followed by 10 cycles of 95°C for 45 s, 40°C for 45 s, and 72°C for 1 min, and 20 cycles of 95°C for 45 s, 45°C for 45 s and 72°C for 1 min. In the second round of PCR, a nested transposon primer (5-PIT2(10868) or Tn5-OE end(5929)-seq) was paired with a primer targeted to the tail portion of the semi-degenerate primer (ARB2 or ARB2A), and 1 μL of PCR product from the first round of PCR was used as a template. For the second round of arbitrary PCR, PCR mix containing 1× GoTaq buffer (Promega), 10% DMSO, 2.5 μM dNTPs, 1.25 units of GoTaq polymerase, 200 nM of each primer, and a 1/10 volume of the first round PCR product as a template was used. Thermal cycling parameters were 40 cycles of 95°C for 45 s, 50°C for 45 s, and 72°C for 1 min, followed by 72°C for 1 min. Samples that produced distinct bands on an agarose gel after the second round of PCR were sequenced with the Tn5-OE end(5929)-seq primer by using BigDye Terminator v3.1 Cycle Sequencing Kit (Thermo Fisher Scientific, MA, USA) and ABI PRISM 310 Genetic Analyzer (Thermo Fisher Scientific). The resulting sequences were subjected to NCBI BLAST search (https://blast.ncbi.nlm.nih.gov/Blast.cgi), and the sequence that exhibited a perfect match with a genomic locus of *P*. *aeruginosa* PAO1 (http://www.pseudomonas.com/) was specified as the transposon insertion site.

**Table 1:** PCR primers used in this study.

Primer name	Sequence (5′ to 3′)
ARB1 [27]	GGCCACGCGTCGACTAGTACNNNNNNNNNNGATAT
ARB2 [27]	GGCCACGCGTCGACTAGTAC
ARB1D [26]	GGCCAGGCCTGCAGATGATGNNNNNNNNNNGTAT
ARB2A [26]	GGCCAGGCCTGCAGATGATG
Tn5-OE end(5929)-arb	ACTTGTGTATAAGAGTCA
Tn5-OE end(5929)-seq	ACTTGTGTATAAGAGTCAG
5-pIT2(10841)	ATCAGATCCCCCTGGATGGA
5-pIT2(10868)	AAAGGTTCCGTCCAGGACGC
5-PA-serA-Hind3-pro-356681	GAGAAAGCTTATCAGGGCTTCGCGGGTCAT
3-PA-serA-Xba1 -end-355248	GAGATCTAGATTAGAACAGCACGCGGCTAC
5-PA-serA-BamH1-ATG-356477	GAGAGGATCCATGAGCAAGACCTCTCTCGA
3-PA-serA-Xho1-end- 355248	GAGACTCGAGTTAGAACAGCACGCGGCTAC

**Table 2:** Arbitrary PCR primers used in this study.

Gene Name	1st PCR		2nd PCR	
	Forward	Reverse	Forward	Reverse
*fruK*	5-PIT2(10841)	ARB1D	5-PIT2(10868)	ARB2A
*dnaK*	Tn5-OE end(5929)-arb	ARB1	Tn5-OE end(5929)-seq	ARB2
*suhB*	Tn5-OE end(5929)-arb	ARB1	Tn5-OE end(5929)-seq	ARB2
*serA*	Tn5-OE end(5929)-arb	ARB1	Tn5-OE end(5929)-seq	ARB2
*aroA*	Tn5-OE end(5929)-arb	ARB1D	Tn5-OE end(5929)-seq	ARB2A
*purL*	Tn5-OE end(5929)-arb	ARB1D	Tn5-OE end(5929)-seq	ARB2A
*aceE*	Tn5-OE end(5929)-arb	ARB1D	Tn5-OE end(5929)-seq	ARB2A
*chpA*	Tn5-OE end(5929)-arb	ARB1	Tn5-OE end(5929)-seq	ARB2
*fimV*	Tn5-OE end(5929)-arb	ARB1D	Tn5-OE end(5929)-seq	ARB2A
*pilB*	Tn5-OE end(5929)-arb	ARB1	Tn5-OE end(5929)-seq	ARB2
*pilC*	Tn5-OE end(5929)-arb	ARB1D	Tn5-OE end(5929)-seq	ARB2A
*pilD*	Tn5-OE end(5929)-arb	ARB1D	Tn5-OE end(5929)-seq	ARB2A
*pilF*	Tn5-OE end(5929)-arb	ARB1	Tn5-OE end(5929)-seq	ARB2
*pilM*	Tn5-OE end(5929)-arb	ARB1D	Tn5-OE end(5929)-seq	ARB2A
*pilQ*	Tn5-OE end(5929)-arb	ARB1	Tn5-OE end(5929)-seq	ARB2
*pilR*	5-PIT2(10841)	ARB1	5-PIT2(10868)	ARB2
*pilS*	Tn5-OE end(5929)-arb	ARB1	Tn5-OE end(5929)-seq	ARB2
*pilV*	5-PIT2(10841)	ARB1D	5-PIT2(10868)	ARB2A
*pilY1*	Tn5-OE end(5929)-arb	ARB1	Tn5-OE end(5929)-seq	ARB2
*flgE*	Tn5-OE end(5929)-arb	ARB1	Tn5-OE end(5929)-seq	ARB2
*flgK*	Tn5-OE end(5929)-arb	ARB1D	Tn5-OE end(5929)-seq	ARB2A

To construct the plasmid used for *serA* complementation, a 1433-bp *Hind*III-*Xba*I fragment carrying the *P*. *aeruginosa SerA* ORF was amplified by PCR using 5-PA-serA-Hind3-pro-356681 and 3-PA-serA-Xba1-end-355248 primers; this insert corresponds to nucleotides 355248 to 356681 in the PAO1 genome sequence (www.pseudomonas.com). The *Hind*III-*Xba*I fragment was cloned into the *Hind*III-*Xba*I site of pUCP19 [28], which was kindly provided by Dr. Colin Manoil (University of Washington), and the resultant plasmid was designated pUCP19-*serA*. The pUCP19-*serA* plasmid was electroporated into PAO1Tn::*serA*, and the resulting transformant was designated PAO1Tn::*serA* (pUCP19-*serA*).

### Secretion assay of ExoS

Secretion of type III effector ExoS can be induced *in vitro* by removing calcium from the medium [29]. A secretion assay was performed as previously described [29] with a slight modification. Bacteria were diluted 1:300 into “high-salt” LB (medium containing 200 mM NaCl, 10 mM MgCl_2_, and 0.5 mM CaCl_2_) and grown for 2 h, at which point secretion was induced through the addition of EGTA (5 mM, final concentration). The cultures were allowed to grow for another 2 h, and the bacteria were pelleted by centrifugation. As required, _L_**-**serine was added at the indicated concentrations to the culture. Supernatant proteins were precipitated with 10% (final concentration) trichloroacetic acid (TCA). Pellets were washed three times with acetone and resuspended in phosphate buffered saline (PBS). After determining the protein concentration with a BCA Protein Assay Reagent kit (Thermo Fisher Scientific), samples (8 μg protein) were separated by SDS-PAGE, transferred to an Amersham Protran 0.45-μm nitrocellulose membrane (GE Healthcare), and probed with a rabbit anti-ExoS polyclonal antibody. Anti-ExoS polyclonal antibody was prepared as previously described [30].

### Bacterial adherence to Caco-2 cells

Bacterial adherence to Caco-2 cells was performed as previously described [31] with a slight modification. Caco-2 cells were seeded and grown on sterile glass coverslips in four-well tissue culture plates (Nunc Lab-Tek II Chamber Slide System, Thermo Fisher Scientific). For the adherence assays, bacteria were grown to mid-log growth phase in LB broth and collected by centrifugation and diluted in Hank’s Balanced Salt Solution (HBSS) plus supplements (1 mM CaCl_2_, 2 mM MgCl_2_, and 20 mM HEPES). Bacteria were introduced at a MOI of 100 bacteria per Caco-2 cell and the infection was allowed to proceed for 1 h at 37°C in 5% CO_2_. As needed, _L_**-**serine was added at the indicated concentrations to Caco-2 cells. The Caco-2 cells were washed four times with 1 mL of HBSS plus supplements to remove unattached bacteria. Co-cultures were fixed with 2.5% glutaraldehyde in PBS overnight at 4°C and then in 5% formaldehyde, 5% glacial acetic acid, and 70% methanol for 1 h, followed by staining with Giemsa stain for 10 min. Coverslips were mounted on glass slides and viewed by light microscopy at 1000× magnification. Eukaryotic cells and associated bacteria from six fields, representing different regions of the coverslip, were enumerated and reported as the number of bacteria per Caco-2 cell.

### Motility assays

Swarming motility and swimming motility assays were performed as previously described [32,33] with a slight modification. Swarm motility plates (0.5% agar) and swim motility plates (0.3% agar) contained M8 medium supplemented with 1 mM MgSO4, 0.2% glucose, and 0.5% CAA. LB overnight cultures (2 μL) were inoculated onto the surface of the swarm and swim plates and were incubated for 12 h at 37°C and for 14 h at 30°C, respectively. As required, _L_**-**serine was added at the indicated concentrations to the swarm and swim plates. The major axes of swarming and swimming were measured.

Twitching motility assays were performed as previously described [34] with a slight modification. Twitch motility plates (1.5% agar) were comprised of M8 medium supplemented with 1 mM MgSO4, 0.2% glucose, and 0.5% CAA. Twitch plates were dried and strains were stab-inoculated with a toothpick to the bottom of the Petri dish using an LB agar plate that was grown overnight. After incubation for 24 h at 37°C, the resulting zones of twitching motility were visualized by carefully removing the agar and staining bacteria that adhered to the polystyrene Petri plate with 1% crystal violet for 10 min at room temperature. This was followed by a brief rinse with tap water to remove the unbound dye. The major axes of twitching were measured.

### Staining of bacterial flagella

Staining of bacterial flagella was performed as previously described [35] with a slight modification. Surface colonies grown on an LB agar plate were suspended directly in distilled water on the slides. After drying the slides, the slides were stained with the Leifson stain (1% tannic acid, 0.5% sodium chloride, 0.3% pararosaniline acetate, 0.1% pararosaniline hydrochloride, and 32% ethanol) for 10 min, and the slides were observed at 1000× magnification after washing.

### PGDH activity assay

To construct the plasmid used for *serA* expression, *Bam*HI-*Xho*I fragment carrying *P*. *aeruginosa serA* ORF was amplified by PCR using 5-PA-serA-*Bam*HI-ATG-356477 and 3-PA-serA-*Xho*1-end-355248 primers; this insert corresponds to the nucleotides 355248 to 356477 in the PAO1 genome sequence (www.pseudomonas.com). The *Bam*HI-*Xho*I fragment was ligated into the *Bam*HI-*Sal*I site of ptac-85 [36], and the resultant plasmid (ptac-85-serA) was transformed into *E*. *coli* DH5α.The resulting transformant was designated DH5α (ptac-85-*serA*). As a negative control (mock) strain, ptac-85 vector was transformed into *E*. *coli* DH5α, and the resulting transformant was designated DH5α (ptac-85).

The crude extract containing the overexpressed *P*. *aeruginosa* SerA protein was isolated from the DH5α (ptac-85-*serA*) culture as previously described [19,37] with a slight modification. A 20 mL aliquot of DH5α (ptac-85-*serA*) culture or DH5α (ptac-85) mock culture grown in LB broth supplemented with 100 μg/mL of ampicillin was inoculated into 200 mL of fresh LB broth supplemented with 100 μg/mL of ampicillin, incubated at 37°C for 3 h, and SerA expression was then induced with 1 mM isopropyl-β-D-thiogalactopyranoside and allowed to grow at 37°C for 4 h. The cells were harvested by centrifugation at 4,000 ×*g* for 5 min and resuspended in 20 mM potassium phosphate (pH 7.5) containing 5 mM KCl. After addition of lysozyme (0.16 mg/mL), the cells were disrupted by sonication for 2.5 min. After the cell debris was removed by centrifugation at 20,000 ×*g* for 10 min, sodium chloride was added to the supernatant at a final concentration of 100 mM. Protein quantitation was performed using Pierce BCA Protein Assay Kit (Thermo Fisher Scientific). The crude extract (10 μg) was applied to 12% SDS-PAGE gel analysis, after which proteins were stained using Rapid Stain CBB Kit (Nacalai Tesque).

PGDH activity assay in the crude extract was carried out as previously described [37] with a modification. Briefly, PGDH activity was measured in the crude extract by detecting a decrease in absorbance at 339 nm in the presence of NADH (Sigma-Aldrich, USA) and hydroxypyruvic acid phosphate (HPAP; Sigma-Aldrich). The assays were performed in five replicates in 200 mM potassium phosphate buffer (pH 7.5) containing 250 μM NADH, 350 μM HPAP, and 50 mM _L_-serine where necessary. The enzyme activity (unit per mg protein) was calculated by following the enzyme unit reported previously [38, 39].

### Fly survival experiments

Fly survival was evaluated as previously described [20,21] with a modification. *D*. *melanogaster* flies (7–10 days old) were purchased from Sumika Technoservice Corporation (Japan). Twenty-four to thirty flies in a plastic container were fed a 5% sucrose suspension containing LB overnight cultures of each *P*. *aeruginosa* strain on a sterile paper disc. As needed, _L_**-**serine was added at the indicated concentrations, to the 5% sucrose suspension containing the *P*. *aeruginosa* culture on a sterile paper disc. The flies were reared on the bacterium-sucrose mixture for the duration of the experiment. Flies were maintained at 25°C and survival was monitored daily.

### Statistical analysis

Statistical analysis, except for fly survival analysis, was performed using a two-tailed *t* test. For fly survival analysis, the Log-Rank test was applied for testing differences between the survival curves, and the statistical analysis software EZR [40] was used.

## Results

### Mutant screening of penetration activity

**Fig 1:**
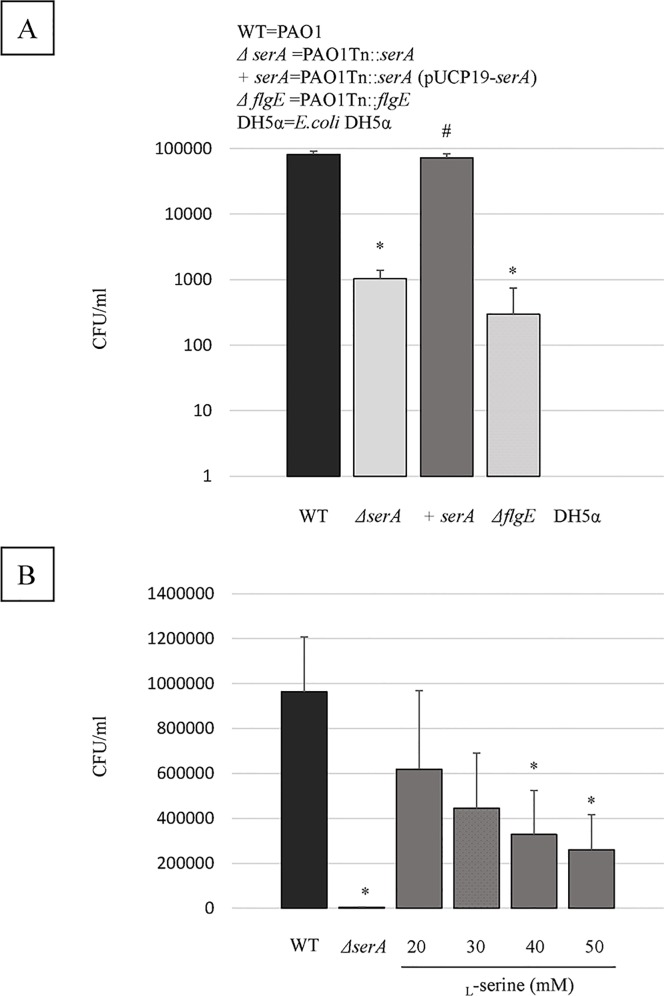
Penetration activity of *P*. *aeruginosa* strains. (A) The wild-type strain (WT), PAO1Tn::*serA* mutant (*ΔserA*), PAO1Tn::*serA* (pUCP19-*serA*) complementary strain (+*serA*), and PAO1Tn::*flgE* mutant (*ΔflgE*) were inoculated onto the apical surfaces of Caco-2 cell monolayers at an MOI of 100, and the number of bacteria in the basolateral medium was counted at 6 h after infection. The assay was performed in triplicate, and the results are expressed as the mean ± SD. *E*. *coli* DH5α was used as a negative control. *#: P < 0.05; *: vs WT; #: vs Δ*serA* (B) Influence of _L_-serine addition on penetration activity of the wild-type strain. The assay was performed in triplicate, and the results are expressed as mean ± SD. *: P < 0.05; *: vs WT.

To specify genes associated with bacterial translocation through the intestinal epithelial cell monolayer, we screened 3070 transposon insertion mutants for reduced penetration through Caco-2 cell monolayers, when compared to the penetration by the wild-type strain. This investigation identified 62 mutants, from which 21 genes were specified through the analysis of transposon insertion sites using a two-round arbitrary PCR protocol (Table 3). Among these 21 genes, we focused our attention on the *serA* gene (PA0316), as it is not known how PGDH contributes to the ability of *P*. *aeruginosa* to penetrate the intestinal epithelial cell barrier. There was no significant difference in the growth of the wild-type strain and PAO1Tn::*serA* mutant at 24 h after incubation in LB broth (S2 Fig). Subsequently, we performed penetration assays using PAO1, PAO1Tn::*serA* and complementary PAO1Tn::*serA* (pUCP19-*serA*) strains. We infected Caco-2 cell monolayers with these variants to determine if the *serA* gene is necessary for epithelial monolayer penetration in this strain. As shown in Fig 1A, penetration of the PAO1Tn::*serA* strain through Caco-2 cell monolayers showed a 98.7% reduction compared with the PAO1 strain at 6 h after infection, and the difference was statistically significant (P < 0.05). The penetration activity of the PAO1Tn::*serA* strain was almost identical to that of a flagellar hook protein mutant, PAO1Tn::*flgE* (Δ*flgE*) (Fig 1A). The decrease in the ability of PAO1Tn::*serA* to penetrate the monolayers was largely restored in the PAO1Tn::*serA* (pUCP19-*serA*) complemented strain (Fig 1A; P < 0.05).

**Table 3:** Specification of genes responsible for penetration ability.

locus_tag	Homolog in database	Gene Name	GenBank accession no.	No. of clones isolated
PA3561	1-phosphofructokinase	*fruK*	NP_252251.1	1
PA4761	heat shock protein DnaK	*dnaK*	NP_253449.1	1
PA3818	extragenic suppressor protein SuhB	*suhB*	NP_252507.1	1
PA0316	D-3-phosphoglycerate dehydrogenase	*serA*	NP_249007.1	1
PA3164	still frameshift 3-phosphoshikimate 1-carboxyvinyltransferase	*aroA*	AE_004091.2	2
PA3763	phosphoribosylformylglycinamidine synthase	*purL*	NP_252452.1	1
PA5015	pyruvate dehydrogenase	*aceE*	NP_253702.1	3
PA0413	component of chemotactic signal transduction system	*chpA*	NP_249104.1	2
PA3115	motility protein FimV	*fimV*	NP_251805.1	2
PA4526	type 4 fimbrial biogenesis protein PilB	*pilB*	NP_253216.1	1
PA4527	still frameshift type 4 fimbrial biogenesis protein PilC	*pilC*	NP_253217	1
PA4528	type 4 prepilin peptidase PilD	*pilD*	NP_253218.1	1
PA3805	type 4 fimbrial biogenesis protein PilF	*pilF*	NP_252494.1	1
PA5044	type 4 fimbrial biogenesis protein PilM	*pilM*	NP_253731.1	2
PA5040	type 4 fimbrial biogenesis outer membrane protein PilQ precursor	*pilQ*	NP_253727.1	1
PA4547	two-component response regulator PilR	*pilR*	NP_253237.1	1
PA4546	two-component sensor PilS	*pilS*	NP_253236.1	3
PA4551	type 4 fimbrial biogenesis protein PilV	*pilV*	NP_253241.1	1
PA4554	type 4 fimbrial biogenesis protein PilY1	*pilY1*	NP_253244.1	3
PA1080	flagellar hook protein FlgE	*flgE*	NP_249771.1	1
PA1086	flagellar hook-associated protein 1 FlgK	*flgK*	NP_249777.1	2

### Inhibitory effect of _L_-serine on PAO1 penetration through Caco-2 cell monolayers

We investigated whether the addition of _L_-serine could affect penetration of PAO1 through Caco-2 cell monolayers. As shown in Fig 1B, 40 and 50 mM of _L_-serine significantly reduced the penetration of PAO1.

### Type III effector secretion

**Fig 2:**
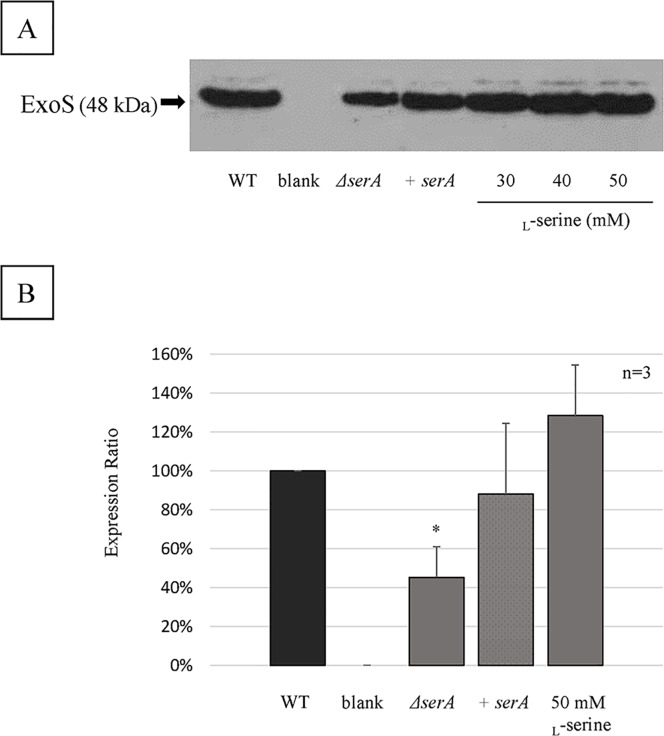
ExoS secretion assay. (A) Western blot analysis to detect secretion of ExoS into the culture supernatant using type III secretion system-inducing conditions in the wild-type strain (WT), PAO1Tn::*serA* mutant (*ΔserA*), PAO1Tn::*serA* (pUCP19-*serA*) complementary strain (+*serA*), and WT in the presence of _L_-serine (30, 40, and 50 mM). A representative western blot image is shown. The arrow indicates the presence of ExoS with a deduced molecular weight of 48 kDa. (B) Expression ratio of ExoS based on western blot analyses of WT, *ΔserA*, +*serA*, and WT in the presence of 50 mM _L_-serine. Western blotting was repeated three times, and bands were quantified by ImageJ. Data is shown as the ratio of ExoS expression to that of the wild-type strain and is expressed as the mean ± SD. A significant difference was observed for ExoS expression between WT and *ΔserA* (*: P < 0.05).

As shown in Fig 2, secretion of ExoS in the culture supernatant was significantly repressed in the PAO1Tn::*serA* mutant compared to that in the wild-type strain, and complementation of the *serA* gene in the PAO1Tn::*serA* mutant resulted in recovery of ExoS secretion. However, the addition of _L_-serine (30 to 50 mM) did not affect secretion of ExoS in the wild-type strain.

### Staining of bacterial flagella

**Fig 3:**
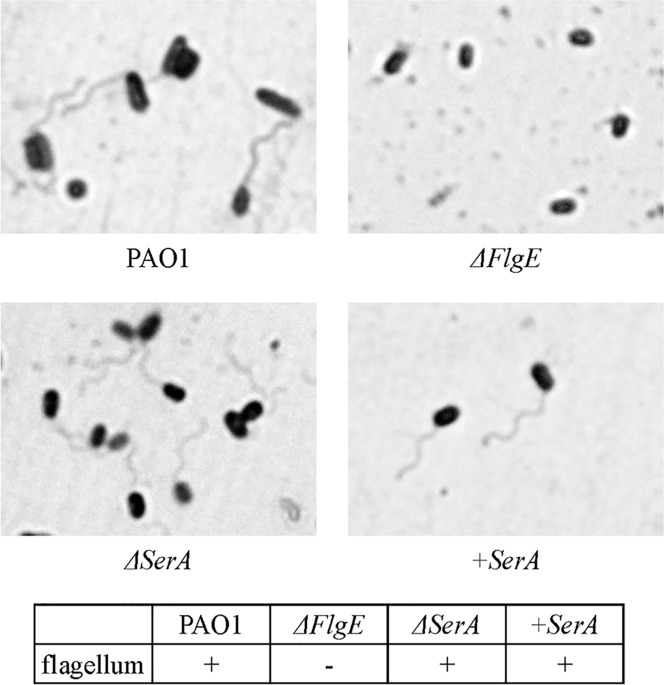
Staining of bacterial flagella. Flagella staining of the wild-type strain (WT), PAO1Tn::*serA* mutant (*ΔserA*), PAO1Tn::*serA* (pUCP19-*serA*) complementary strain (+*serA*), and PAO1Tn::*flgE* mutant (*ΔflgE*) was performed as previously described [35].

As shown in Fig 3, bacterial flagella were observed in the PAO1Tn::*serA* mutant as well as the wild-type strain and the PAO1Tn::*serA* (pUCP19-*serA*) complementary strain, but not in the *ΔflgE* strain, which was used as a negative control.

### Motility assays

**Fig 4:**
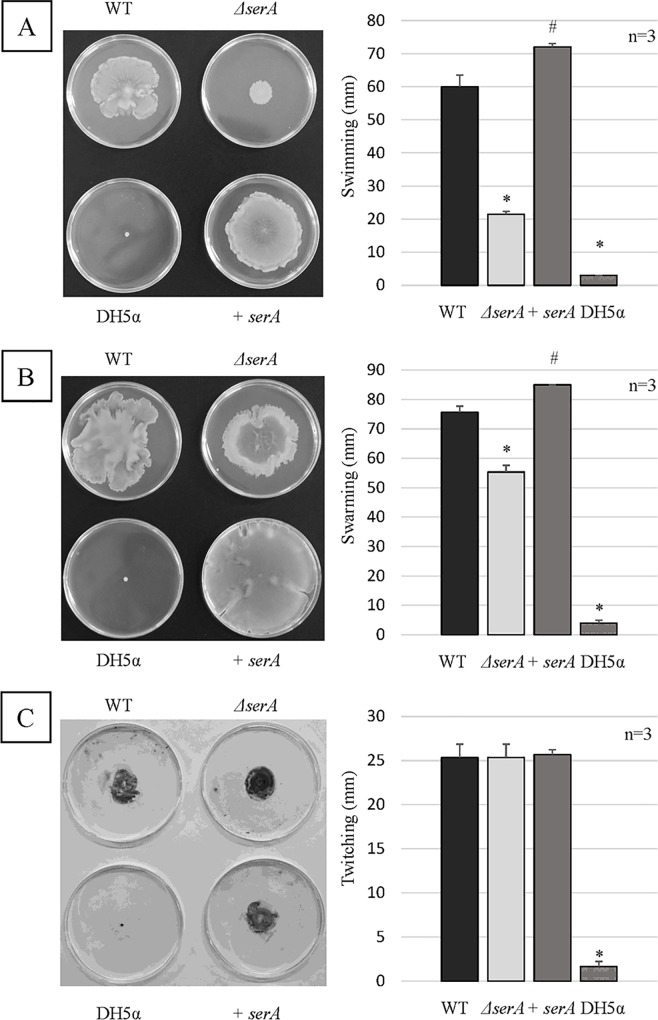
Motility assays. (A) Swimming motility of WT, *ΔserA*, +*serA*, and *E*. *coli* DH5α (as a negative control). A representative image from the swimming motility assay is shown. The major axis of swimming is the longest length of the swimming area. The assay was performed in triplicate, and the results are expressed as the mean ± SD. Significant differences were observed between WT and *ΔserA* (*: P < 0.05), between WT and +*serA* (^#^: P < 0.05), and between WT and DH5α (*: P < 0.05). (B) Swarming motility in the wild-type strain (WT), PAO1Tn::*serA* mutant (*ΔserA*), PAO1Tn::*serA* (pUCP19-*serA*) complementary strain (+*serA*), and *E*. *coli* DH5α (as a negative control). A representative image of the swarming motility assay is shown. The major axis of swarming is the longest length of the swarming area. The assay was performed in triplicate, and the results are expressed as the mean ± SD. Significant differences were observed between WT and *ΔserA* (*: P < 0.05), between WT and +*serA* (^#^: P < 0.05), and between WT and DH5α (*: P < 0.05). (C) Twitching motility in WT, *ΔserA*, +*serA*, and *E*. *coli* DH5α (as a negative control). A representative image of the twitching motility assay is shown. The major axis of twitching is the longest length of the twitching area. The assay was performed in triplicate, and the results are expressed as the mean ± SD. Significant difference was observed between WT and DH5α (*: P < 0.05).

Swimming motility of the PAO1Tn::*serA* mutant was greatly reduced compared with that of the wild-type strain, and complementation with the *serA* gene in the PAO1Tn::*serA* mutant resulted in the recovery of swimming motility (Fig 4A). Next, swarming motility in the PAO1Tn::*serA* mutant was also significantly reduced compared with that in the wild-type strain. However, in the PAO1Tn::*serA* mutant, the degree to which swarming motility was reduced was much lower than that of swimming motility (Fig 4B). Complementation with the *serA* gene in the PAO1Tn::*serA* mutant resulted in the recovery of swarming motility (Fig 4B). In contrast, there was no significant difference in twitching motility between the PAO1Tn::*serA* mutant and the wild-type strain (Fig 4C).

### Inhibitory effect of _L_-serine on swimming and swarming motilities

**Fig 5:**
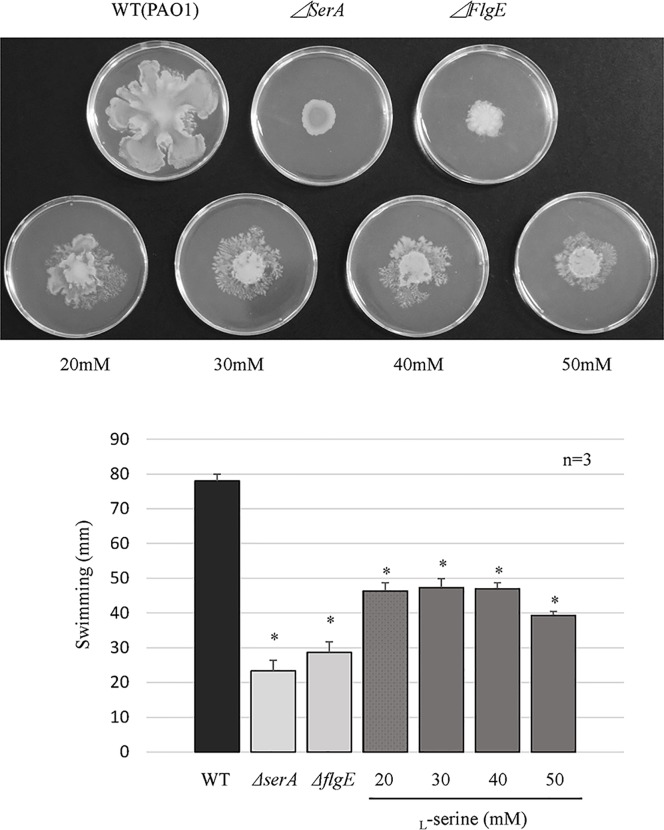
Influence of _L_-serine addition on swimming motility in the wild-type strain. Swimming motility in the wild-type strain (WT), PAO1Tn::*serA* mutant (*ΔserA*), PAO1Tn::*flgE* mutant (*ΔflgE*) (as a negative control), and WT in the presence of _L_-serine (20, 30, 40, and 50 mM). A representative image from the swarming motility assay is shown. The major axis of swimming is the longest length of the swimming area. The assay was performed in triplicate, and the results are expressed as the mean ± SD. Significant differences were observed between WT and *ΔserA* (*: P < 0.05), between WT and *ΔflgE* (*: P < 0.05), between WT and WT in the presence of 20 mM _L_-serine (*: P < 0.05), between WT and WT in the presence of 30 mM _L_-serine (*: P < 0.05), between WT and WT in the presence of 40 mM _L_-serine (*: P < 0.05), and between WT and WT in the presence of 50 mM _L_-serine (*: P < 0.05).

**Fig 6:**
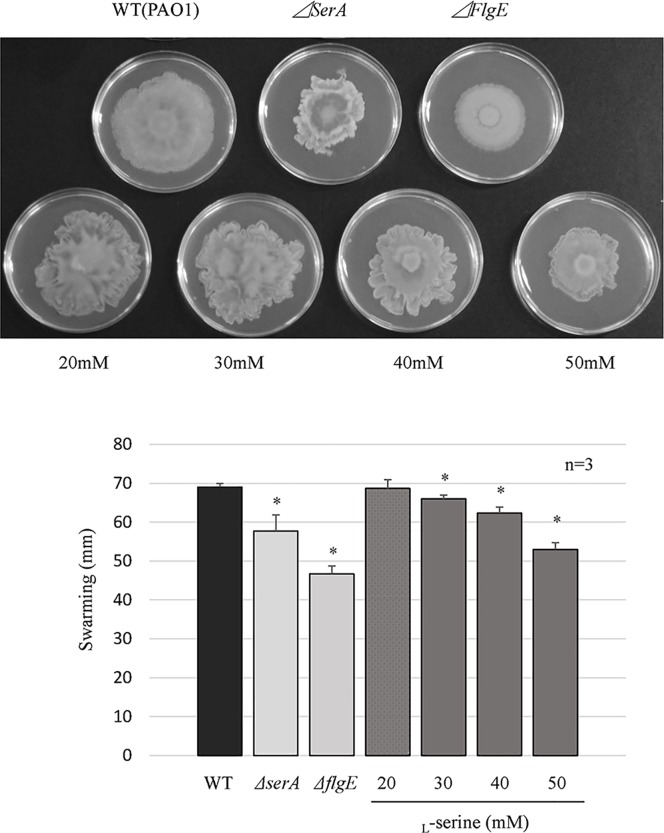
Influence of _L_-serine addition on swarming motility in the wild-type strain. Swarming motility of the wild-type strain (WT), PAO1Tn::*serA* mutant (*ΔserA*), PAO1Tn::*flgE* mutant (*ΔflgE*) (as a negative control), and WT in the presence of _L_-serine (20, 30, 40, and 50 mM). A representative image of the swarming motility assay is shown. The major axis of swarming is the longest length of the swarming area. The assay was performed in triplicate, and the results are expressed as the mean ± SD. Significant differences were observed between WT and *ΔserA* (*: P < 0.05), between WT and *ΔflgE* (*: P < 0.05), between WT and WT in the presence of 30 mM _L_-serine (*: P < 0.05), between WT and WT in the presence of 40 mM _L_-serine (*: P < 0.05), and between WT and WT in the presence of 50 mM _L_-serine (*: P < 0.05).

Deletion of *serA* resulted in a significant reduction in swimming and swarming motilities. We then investigated if the addition of _L_-serine could influence both types of motility. As shown in Fig 5, the addition of 20 to 50 mM of _L_-serine, in the culture medium, significantly suppressed swimming motility in the wild-type strain. However, the degree to which swimming motility was reduced through the addition of _L_-serine was lower than that of the PAO1Tn::*serA* mutant and *ΔflgE* strain (used as a negative control). As shown in Fig 6, the addition of 30 to 50 mM of _L_-serine to the medium also significantly repressed swarming motility in the wild-type strain and to an extent similar to that observed for the PAO1Tn::*serA* and *ΔflgE* mutants.

### Inhibitory effect of *serA* mutation and _L_-serine on Caco-2 adherence

**Fig 7:**
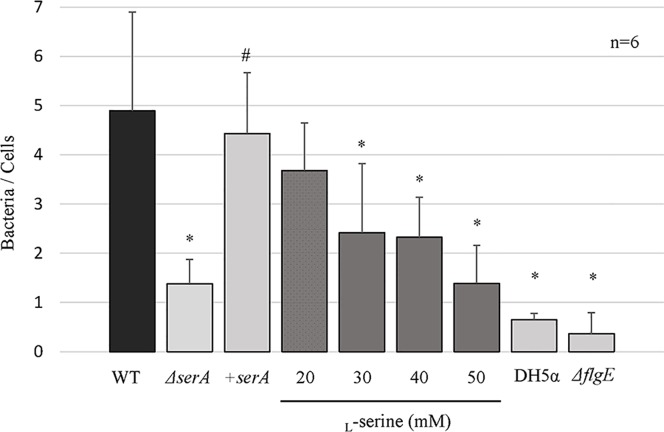
Bacterial adherence to Caco-2 cells for the wild-type strain (WT), PAO1Tn::*serA* mutant (*ΔserA*), PAO1Tn::*serA* (pUCP19-*serA*) complementary strain (+*serA*), PAO1Tn::*flgE* mutant (*ΔflgE*), *E*. *coli* DH5α (as a negative control), and WT in the presence of _L_-serine (20, 30, 40, and 50 mM). Bacterial adherence was determined based on the number of adhered bacteria per Caco-2 cell. The assay was performed in six replicates, and the results are expressed as the mean ± SD. Significant differences were observed between WT and *ΔserA* (*: P < 0.05), between WT and DH5α (*: P < 0.05), between WT and *ΔflgE* (*: P < 0.05), between WT and WT in the presence of 30 mM _L_-serine (*: P < 0.05), between WT and WT in the presence of 40 mM _L_-serine (*: P < 0.05), and between WT and WT in the presence of 50 mM _L_-serine (*: P < 0.05). Bacterial adherence of the +*serA* complementary strain was significantly restored as compared to that of *ΔserA* (#; P < 0.05).

Bacterial adherence to the Caco-2 cells of the PAO1Tn::*serA* mutant exhibited a 71.8% reduction compared to that of the wild-type strain, and the difference was statistically significant (Fig 7). Furthermore, complementation with the *serA* gene in the PAO1Tn::*serA* mutant resulted in the recovery of this phenotype (Fig 7). In addition, bacterial adherence to Caco-2 cells with *E*. *coli* DH5α showed an 86.7% reduction compared to that of the wild-type strain, as previously reported [12], and the difference was statistically significant (Fig 7). Adherence of *ΔflgE* mutant strain to the Caco-2 cells also exhibited a 92.6% reduction compared to that of the PAO1 wild-type strain, and the difference was statistically significant. The reduction in bacterial adherence was similar between *ΔflgE* and *E*. *coli* DH5α (negative control) (Fig 7).

We next investigated whether the addition of _L_-serine could influence Caco-2 bacterial adherence. As shown in Fig 7, the addition of 30 to 50 mM of _L_-serine significantly repressed the adherence of the wild-type strain to Caco-2 cells, to almost the same extent as that observed for the PAO1Tn::*serA* mutant.

### PGDH activity of the SerA protein

**Fig 8:**
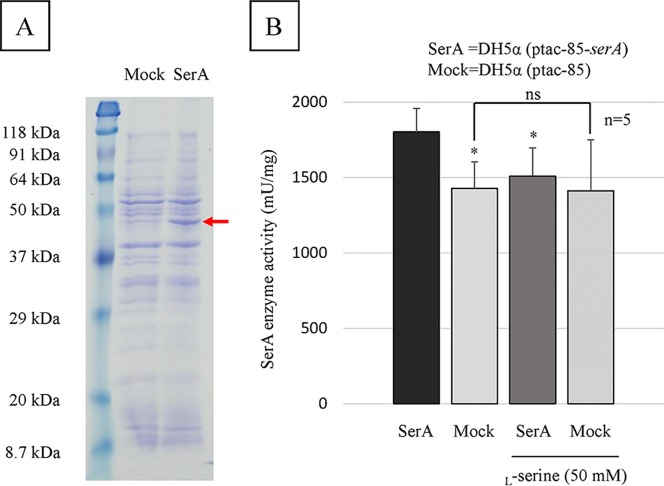
PGDH activity assay. (A) SDS-PAGE analysis to verify overexpression of the *P*. *aeruginosa serA* and synthesis of SerA proteins in crude extract isolated from DH5α (ptac-85-*serA*) culture (SerA) and from DH5α (ptac-85) culture (mock). The red arrow indicates an overexpressed band at the expected size of 44 kDa for *P*. *aeruginosa* SerA. (B) Inhibitory effect of 50 mM _L_-serine on PGDH activity of SerA proteins (mU/mg) in crude extract isolated from DH5α (ptac-85-*serA*) culture (SerA) and from DH5α (ptac-85) culture (mock). The assay was performed in five replicates, and the results are expressed as the mean ± SD. Significant differences were observed between SerA and mock (*: P < 0.05), and between SerA and SerA in the presence of 50 mM _L_-serine (*: P < 0.05). There was no significant difference between mock and mock in the presence of 50 mM _L_-serine (ns: P > 0.9).

To verify overexpression of the *P*. *aeruginosa serA* and synthesis of SerA proteins, crude extracts were analyzed by SDS-PAGE. An overexpressed band at the expected size of 44 kDa for *P*. *aeruginosa* SerA could be detected from the crude extract isolated from DH5α (ptac-85-*serA*) culture as compared with that isolated from DH5α (ptac-85) mock culture (Fig 8A).

We then investigated whether _L_-serine has the ability to directly inhibit PGDH activity of the SerA protein. The SerA enzyme activity (mU/mg) of the crude extract isolated from DH5α (ptac-85-*serA*) culture was 26% higher than that isolated from DH5α (ptac-85) mock culture, and the difference was statistically significant (P < 0.05) (Fig 8B). Furthermore, addition of _L_-serine (50 mM) caused a 16% reduction of the SerA enzyme activity in the crude extract isolated from DH5α (ptac-85-*serA*) culture, and the difference was statistically significant (P < 0.05) (Fig 8B), whereas addition of _L_-serine (50 mM) did not result in a significant reduction of the SerA enzyme activity in the crude extract isolated from DH5α (ptac-85) mock culture (Fig 8B). Therefore, these results suggest that the phenotypes seen with the addition of _L_-serine are through inhibition of SerA PGDH activity.

### Association between *serA* and PAO1 virulence in flies and contribution of _L_-serine to protection from PAO1 infection

**Fig 9:**
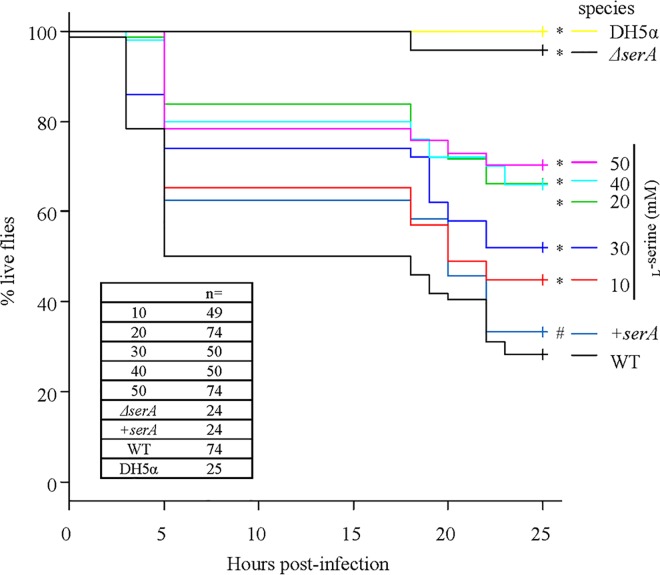
Fly survival experiments. Virulence of the wild-type strain (WT), PAO1Tn::*serA* mutant (*ΔserA*), PAO1Tn::*serA* (pUCP19-*serA*) complementary strain (+*serA*), *E*. *coli* DH5α (as a negative control), and WT in the presence of _L_-serine (10, 20, 30, 40, and 50 mM) was evaluated. Significant differences, based on the log-rank test, were observed between WT and DH5α (*: P < 0.05), between WT and *ΔserA* (*: P < 0.05), between WT and WT in the presence of 50 mM _L_-serine (*: P < 0.05), between WT and WT in the presence of 40 mM _L_-serine (*: P < 0.05), between WT and WT in the presence of 20 mM _L_-serine (*: P < 0.05), between WT and WT in the presence of 30 mM _L_-serine (*: P < 0.05), and between WT and WT in the presence of 10 mM _L_-serine (*: P < 0.05). Virulence of the +*serA* complementary strain was significantly restored as compared to that of *ΔserA* (#; P < 0.05).

The virulence of the PAO1Tn::*serA* mutant was significantly attenuated compared to that of the wild-type strain, and *serA* complementation in mutant bacteria resulted in an increased mortality rate, to levels associated with the wild-type strain (Fig 9). This suggests that *serA* is required for *P*. *aeruginosa* virulence in flies. Therefore, we hypothesize that the high mortality rate observed in flies following oral infection of the wild-type strain is dependent upon the ability to efficiently penetrate the midgut barrier, which is dependent on the *serA* gene.

Based on the penetration assay, 40 mM and 50 mM of _L_-serine could significantly reduce penetration by the wild-type strain due to _L_-serine-mediated repression of swimming and swarming motilities. Thus, we investigated the inhibitory effect of _L_-serine on virulence in flies using the wild-type strain. As shown in Fig 9, 10 to 50 mM of _L_-serine significantly decreased the mortality rate of the wild-type strain. Together, these findings suggest that *serA* might have a key role in *P*. *aeruginosa* pathogenesis by increasing bacterial penetration of the midgut epithelial cell barrier through the induction of swimming and swarming motility, but not twitching motility.

## Discussion

To clarify the unknown mechanism of *P*. *aeruginosa* penetration through the intestinal barrier, we isolated genes associated with this phenotype using a Tn-insertion mutant library. As a result, we specified 21 types of genes classified as flagellin-associated, pili-associated, heat-shock protein genes, genes related to the glycolytic pathway, and biosynthesis-related genes (Table 3). We focused on *serA*, which, until now, had not been associated with bacterial penetration activity. Inactivation of the *serA* gene caused significant repression of bacterial penetration through Caco-2 cell monolayers, with accompanying decreases in swimming and swarming motilities, bacterial adherence, ExoS secretion, and fly mortality rates; however, twitching motility was not affected. We recently reported that the swarming and swimming motilities mediated by *wecC* and *fliF* genes were essential for the penetration of *Edwardsiella tarda* through Caco-2 cell monolayers [41]. It is thought that flagella also play a key role in the penetration of *P*. *aeruginosa* through the Caco-2 cell monolayers. Flagella-driven swimming and swarming motilities are necessary for *P*. *aeruginosa* to reach the epithelial cells, after which *P*. *aeruginosa* flagella will also act as a tether for the initial adherence to the epithelial cell membranes, as previously described [42]. This was confirmed in this study as bacterial adherence to Caco-2 cells in PAO1Tn::*serA* and PAO1Tn::*flgE* mutants was significantly decreased compared to that of the wild-type strain (Fig 7). As described previously, flagella can function as adhesins to bind epithelial membranes; purified flagellin binds to glycolipids, and particularly to the common membrane constituent GM1 [42,43]. Flagella can tether the bacterium to an exposed site with an accessible GM1 or asialoGM1 moiety as a very early event in establishing the nidus of infection, as described by Feldman et al. In the PAO1Tn::*serA* mutant, flagella were observed by conventional staining as shown in Fig 3, similar to that observed in the wild-type strain. This suggests that impaired swimming and swarming motilities mediated by impaired flagella are sufficient to reduce penetration ability and decrease adherence to Caco-2 cells, as observed in the PAO1Tn::*flgE* mutant that lacks flagella. However, further studies are required to determine whether flagella of the wild-type strain can actually tether the bacterium to an exposed site with an accessible GM1 or asialoGM1 moiety in Caco-2 cell monolayers.

Deletion of *serA* resulted in a significant reduction in swimming and swarming motilities. However, the degree to which swarming motility was reduced was much lower than that of swimming motility in the PAO1Tn::*serA* mutant (Fig 4A and 4B). Furthermore, the addition of _L_-serine significantly influenced both types of motility, but the degree of inhibition induced by _L_-serine on swarming motility was much lower than that on swimming motility (Figs 5 and 6). Although there were significant differences in swarming motility between PAO1 and the PAO1Tn::*serA* mutant and after _L_-serine addition, the statistical significance may not reflect the biological significance; the contribution of swarming motility to the PAO1 penetration ability through Caco-2 cell monolayers may be much lower than that of swimming motility. Further studies are required to clarify this phenomenon.

Bacterial translocation through the epithelial cell barrier within the intestinal tract is likely to be achieved through multiple bacterial functions including bacterial motility for reaching the epithelial cells, adherence to epithelial cells, penetration through the epithelial cell barrier, and survival and growth in the blood after invasion. In the present study, we reveal that the *serA* gene encoding PGDH is associated with virulence in the fly; bacterial translocation through the intestinal cell barrier within the fly intestinal tract, mediated by the *serA* gene, must have resulted in virulence, which was associated with bacterial motility to reach the epithelial cells. However, this is only one of the various steps necessary for *P*. *aeruginosa* to achieve gut-derived sepsis. A number of genes specified in the present Tn-mutant analysis are of unknown function (pertaining to virulence); these are heat-shock protein genes, genes related to the glycolytic pathway, and biosynthesis-related genes, and might also be related to bacterial penetration through the intestinal epithelial cell barrier. Further study will be required to clarify the contribution of these genes to *P*. *aeruginosa* virulence, which will eventually facilitate a more comprehensive understanding of the mechanism of bacterial translocation by *P*. *aeruginosa*.

PGDH from *E*. *coli* and *C*. *glutamicum* are known to catalyze the first committed step in the phosphorylated pathway of _L_-serine biosynthesis, and _L_-serine was shown to inhibit PGDH activity in an allosteric, cooperative manner [17–19]. To confirm the direct inhibition of PGDH activity of *P*. *aeruginosa* SerA by _L_-serine, we performed a PGDH assay using crude extracts that were isolated from overnight culture of *E*. *coli* overexpressing the *P*. *aeruginosa serA* gene, as previously described [19,37]. We confirmed the direct inhibition of PGDH activity through the addition of 50 mM _L_-serine (Fig 8B), although background PGDH activity of the negative control strain was high, presumably due to contamination by unknown proteins in the crude extracts. Therefore, to more clearly confirm the direct inhibition of PGDH activity of *P*. *aeruginosa* SerA by _L_-serine, purification of the SerA protein is needed from overnight culture of *E*. *coli* overexpressing the *serA* gene of the wild-type strain, as previously described [37].

In relation to the type III effector secretion, ExoS secretion in the culture supernatant was significantly repressed by *serA* inactivation (Fig 2). However, _L_-serine did not affect ExoS secretion in the wild-type strain, implying either that _L_-serine affects the bacterial phenotypes independently of SerA or that SerA has additional functions besides PGDH activity. Furthermore, the _L_-serine decreased swimming motility of the wild-type strain, but the colony morphology of the wild-type strain in the presence of _L_-serine was substantially different from that of the PAO1Tn::*serA* mutant (Fig 5). This is another indication that either _L_-serine acts on the bacterial phenotypes independent of SerA, or SerA has additional functions besides PGDH activity. These potential implications should be addressed in future studies.

In this study, we showed that the addition of _L_-serine could suppress bacterial penetration with concomitant reductions in swimming and swarming motilities and bacterial adherence. In addition, fly survival rates were also significantly improved by oral administration of _L_-serine. Based on these results, it is expected that pre-administration of _L_-serine to compromised hosts might result in the prevention of gut-derived sepsis caused by *P*. *aeruginosa*. Further studies using an animal model of endogenous *P*. *aeruginosa* septicemia in neutropenic mice [1] will be required to evaluate this hypothesis. Our ultimate goal is to prevent gut-derived sepsis caused by *P*. *aeruginosa* via administration of a potent inhibitor that could suppress bacterial translocation through the intestinal epithelial cell barrier.

## Supporting Information

S1 Fig Schematic information on arbitrary PCR for specification of the *serA* gene in transposon insertion mutant. Detailed information on arbitrary PCR methodology for identification of mutated gene in transposon insertion mutants is available in internet site: https://pga.mgh.harvard.edu/Parabiosys/projects/host-pathogen_interactions/library_construction.php. Outline of arbitrary PCR methods for specification of the *serA* gene in transposon insertion mutant were as follows: For the first round of arbitrary PCR, a specific primer (Tn5-OE end(5929)-arb) for the transposon sequence was paired with a semidegenerate primer with a defined tail (ARB1). In the second round of PCR, a nested transposon primer (Tn5-OE end(5929)-seq) was paired with a primer targeted to the tail portion of the semidegenerate primer (ARB2). Samples that produced distinct bands on an agarose gel after the second round of PCR were sequenced using the Tn5-OE end(5929)-seq primer. The gene sequence obtained was subjected to NCBI BLAST search (https://blast.ncbi.nlm.nih.gov/Blast.cgi), and the gene sequence exhibited perfect match to the serA gene in the complete genome sequence of *P*. *aeruginosa* PAO1.
(TIF)

S2 Fig Comparison of growth between the wild-type strain and PAO1Tn::*serA* mutant (*ΔserA*). Viable cells of the wild-type strain and *ΔserA* at 24 h after incubation in LB broth were compared. The assay was performed in triplicate, and the results are expressed as the mean ± SD. Significant difference was not observed between WT and *ΔserA* (P > 0.5).
(TIF)
