# Supplementary figures and images for: Pseudomonas aeruginosa serA Gene Is Required for Bacterial Translocation through Caco-2 Cell Monolayers
Source: PLoS One. 2017 Jan 3;12(1):e0169367. doi: 10.1371/journal.pone.0169367 (PMC5207755; doi:10.1371/journal.pone.0169367)

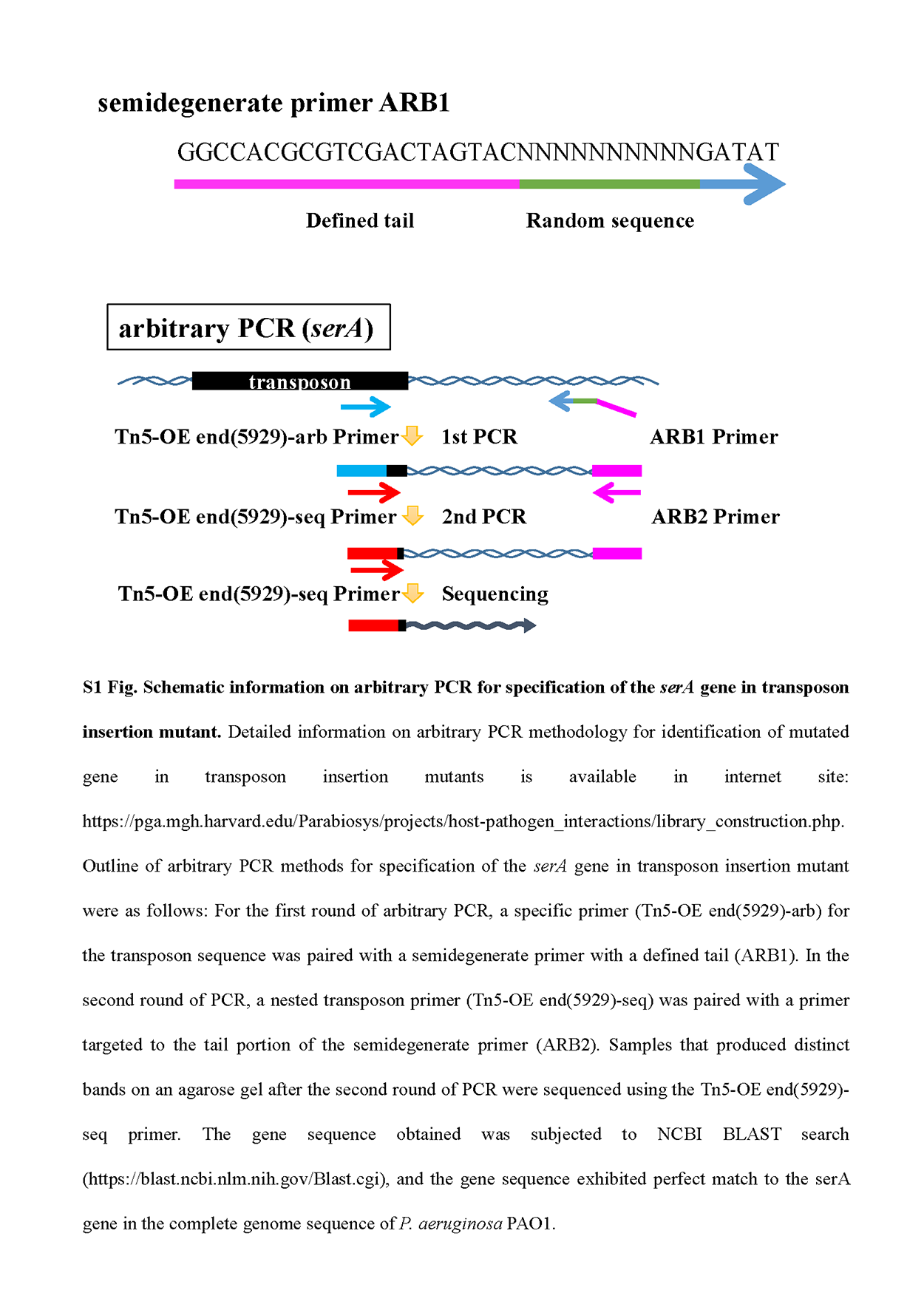

Supplement: S1 Fig — Detailed information on arbitrary PCR methodology for identification of mutated gene in transposon insertion mutants is available in internet site: https://pga.mgh.harvard.edu/Parabiosys/projects/host-pathogen_interactions/library_construction.php. Outline of arbitrary PCR methods for specification of the serA gene in transposon insertion mutant were as follows: For the first round of arbitrary PCR, a specific primer (Tn5-OE end(5929)-arb) for the transposon sequence was paired with a semidegenerate primer with a defined tail (ARB1). In the second round of PCR, a nested transposon primer (Tn5-OE end(5929)-seq) was paired with a primer targeted to the tail portion of the semidegenerate primer (ARB2). Samples that produced distinct bands on an agarose gel after the second round of PCR were sequenced using the Tn5-OE end(5929)-seq primer. The gene sequence obtained was subjected to NCBI BLAST search (https://blast.ncbi.nlm.nih.gov/Blast.cgi), and the gene sequence exhibited perfect match to the serA gene in the complete genome sequence of P. aeruginosa PAO1. (TIF) [file pone.0169367.s001.tif]

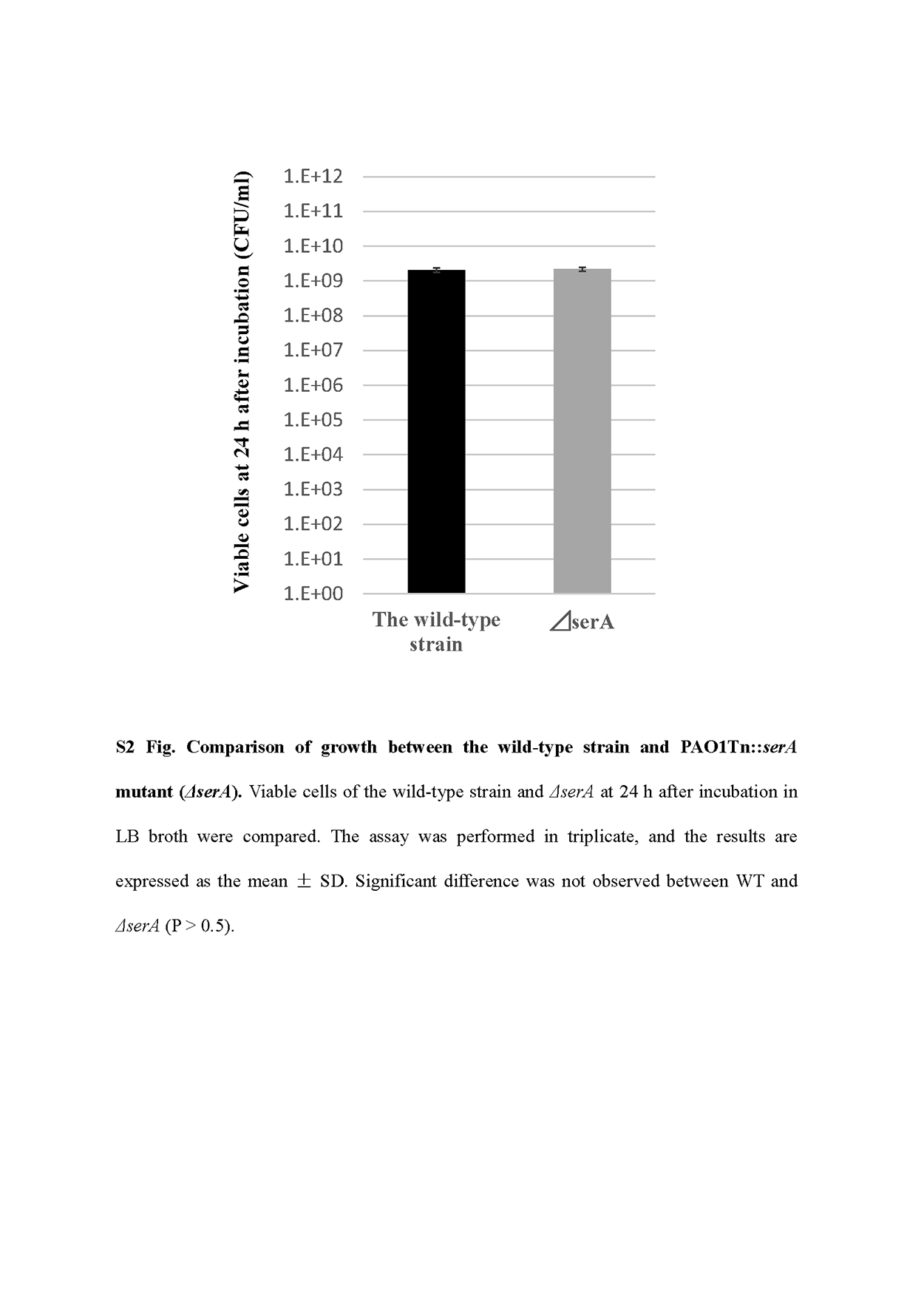

Supplement: S2 Fig — Viable cells of the wild-type strain and ΔserA at 24 h after incubation in LB broth were compared. The assay was performed in triplicate, and the results are expressed as the mean ± SD. Significant difference was not observed between WT and ΔserA (P > 0.5). (TIF) [file pone.0169367.s002.tif]
